# Sensory Prediction of Limb Movement Is Critical for Automatic Online Control

**DOI:** 10.3389/fnhum.2020.549537

**Published:** 2020-10-07

**Authors:** Anne-Emmanuelle Priot, Patrice Revol, Olivier Sillan, Claude Prablanc, Valérie Gaveau

**Affiliations:** ^1^Department of Neurosciences and Cognitive Sciences, Perception Team, Institut de Recherche Biomédicale des Armées, Brétigny-sur-Orge, France; ^2^Lyon Neuroscience Research Center, ImpAct/Trajectoires Team, Bron, France; ^3^Platform ‘Mouvement et Handicap’, Hôpital Henry Gabrielle, Hospices Civils de Lyon, Saint-Genis-Laval, France; ^4^Institute of Rehabilitation Sciences and Techniques (ISTR), Université Claude Bernard Lyon 1, Lyon, France

**Keywords:** motor control, sensory prediction, sensory feedback, multisensory integration, forward internal model

## Abstract

Fast, online control of movement is an essential component of human motor skills, as it allows automatic correction of inaccurate planning. The present study explores the role of two types of concurrent signals in error correction: predicted visual reafferences coming from an internal representation of the hand, and actual visual feedback from the hand. While the role of sensory feedback in these corrections is well-established, much less is known about sensory prediction. The relative contributions of these two types of signals remain a subject of debate, as they are naturally interconnected. We address the issue in a study that compares online correction of an artificially induced, undetected planning error. Two conditions are tested, which only differ with respect to the accuracy of predicted visual reafferences. In the first, “Prism” experiment, a planning error is introduced by prisms that laterally displace the seen hand prior to hand movement onset. The prism-induced conflict between visual and proprioceptive inputs of the hand also generates an erroneous prediction of visual reafferences of the moving hand. In the second, “Jump” experiment, a planning error is introduced by a jump in the target position, during the orienting saccade, prior to hand movement onset. In the latter condition, predicted reafferences of the hand remained intact. In both experiments, after hand movement onset, the hand was either visible or hidden, which enabled us to manipulate the presence (or absence) of visual feedback during movement execution. The Prism experiment highlighted late and reduced correction of the planning error, even when natural visual feedback of the moving hand was available. In the Jump experiment, early and automatic corrections of the planning error were observed, even in the absence of visual feedback from the moving hand. Therefore, when predicted reafferences were accurate (the Jump experiment), visual feedback was processed rapidly and automatically. When they were erroneous (the Prism experiment), the same visual feedback was less efficient, and required voluntary, and late, control. Our study clearly demonstrates that in natural environments, reliable prediction is critical in the preprocessing of visual feedback, for fast and accurate movement.

## Introduction

Everyday movements such as reaching for and grasping a small object, or hitting a moving ball, are subject to various sources of error that originate in bias or noise at either the sensory level or the motor stage. The consequence can be inaccurate movement planning that is not even perceived, but must be corrected. In practice, moderate planning errors are unnoticed as they are corrected by automatic, fast, online processes (Bridgeman et al., 1979; Goodale et al., 1986; Pélisson et al., 1986; Prablanc and Martin, 1992; Desmurget et al., 1999, 2004; Day and Brown, 2001; Desmurget et al., 2001; Sarlegna et al., 2003, 2004; Saunders and Knill, 2003; Gosselin-Kessiby et al., 2008; Gritsenko and Kalaska, 2010; Briere and Proteau, 2011; Oostwoud Wijdenes et al., 2011; Gaveau et al., 2014; Sarlegna and Mutha, 2015; Pruszynski et al., 2016). These corrections are also preemptive to voluntary control and cannot be intentionally suppressed (Day and Lyon, 2000; Pisella et al., 2000).

The role of sensory feedback derived from naturally coherent hand-to-target visual and proprioceptive information in accurate movement correction is well-known (Elliott et al., 2017), in particular, regarding movement variability (Schmidt et al., 1979; Meyer et al., 1982; Desmurget et al., 1995, 1997b). Sensitivity to contextual knowledge has been demonstrated by studies that manipulate not only the probability of perturbation, but also uncertainty about the availability of visual feedback, with a strong emphasis on strategic behavior (Zelaznik et al., 1983; Hansen et al., 2006; Elliott et al., 2010, 2017). Even for natural unperturbed movements the constraints imposed on the movement - such as moving between two points either freely or following a planar path − exhibited large differences in the structure of the movement and in its variability (Desmurget et al., 1997a).

A key challenge in feedback control is the existence of measurable sensory and motor delays. The notion of a *forward model* (Wolpert and Miall, 1996; Kawato and Wolpert, 1998), derived from the former concept of *efference copy* (Von Holst and Mittelstaedt, 1950) was proposed to account for fast processes that are incompatible with sensory processing delays. The forward model’s output, based on weighted, visual and kinesthetic input information from the hand before the movement, enables the central nervous system (CNS) to predict visual reafferences (the visual consequences of motor commands), and cancels the detrimental effect of delays in feedback loops (Wolpert and Miall, 1996; Kawato and Wolpert, 1998).

However, the role of predicted visual reafferences coming from the internal representation of the instantaneous effectors state on fast, online corrections, through a forward internal model, is not fully understood. While prediction error (i.e., the discrepancy between predicted and actual visual reafferences) is known to be an essential component of adaptive behavior (Diedrichsen et al., 2005), the respective roles of online (visual and proprioceptive) feedback and prediction are difficult to assess as both are naturally intermingled. Despite many psychophysical and neurophysiological studies of afferent and efferent contributions to the automatic regulation of motor control, we still lack a full understanding of this sophisticated process (Sarlegna and Mutha, 2015).

The present study attempts to test whether the capability of fast online corrections depends exclusively on sensory feedback control (i.e., on the comparison between perceived goal and actual perceived sensory feedback from the hand: comparison between seen target, and seen and felt moving hand), or whether it also depends on prediction error (i.e., either the comparison between perceived goal and the predicted visual reafferences of the hand, or the comparison between predicted and actual visual feedback from the hand). We address the issue by comparing online correction of an undetected planning error that was generated in two experimental conditions. The latter only differed with respect to the accuracy of predicted visual reafferences (Table 1). In the first (Prism) experiment, a planning error was introduced through prisms that displaced the seen hand prior to movement onset. Planning error is due to an erroneous transformation of visual information about target location to a frame of reference in common with that used to represent the location of the hand in space (for model, McGuire and Sabes, 2009). This prism-induced discrepancy between visual and proprioceptive inputs of the hand also generated an erroneous prediction of hand visual reafferences (Held, 1961). In practice, the initial representation of the hand location is a weighted average of (altered) visual and (non-altered) proprioceptive inputs of the hand before the movement (Rossetti et al., 1995; Ghahramani et al., 1997; Block and Bastian, 2010).

**TABLE 1:** Experimental conditions.

**Perturbation**	**Prism**		**Jump**	
**Visuomotor loop**	**Open loop**	**Closed loop**	**Open loop**	**Closed loop**
Visual feedback prediction	Altered	Altered	Unaltered	Unaltered
Visual feedback during movement	No	Yes	No	Yes
Target vision prior to movement onset	Unaltered	Unaltered	Altered	Altered
Target vision after movement onset	Unaltered	Unaltered	Unaltered	Unaltered
Hand vision prior to movement onset	Altered	Altered	Unaltered	Unaltered
Hand vision after movement onset	No	Yes	No	Yes

In the second (Jump) experiment, a planning error was induced by a jump in the target position during the ocular saccade prior to movement onset. Here, predicted hand reafferences are kept intact as the initial representation of the hand location was not biased. In both Prism and Jump experiments, we either allowed visual feedback from the hand after movement onset (the Closed Loop condition) or removed it (the Open Loop condition). It should be noted that in both experiments, the natural, closed loop condition was designed to induce the same planning errors, eye-hand sequence, movement velocity, and targets. The only difference was the altered or unaltered prediction of visual reafferences after movement onset. In both experimental conditions, similar planning errors were introduced randomly, and were small enough to avoid either strategic behavior or adaptive learning (Magescas and Prablanc, 2006).

If correction depends exclusively on sensory feedback control, correction should be observed when prediction is altered and visual feedback available (closed loop of the Prism experiment). Conversely, if correction also depends on prediction, correction should be incomplete when prediction is altered and visual feedback available. We also predict that unaltered prediction in the jump condition will allow an automatic correction of the artificially introduced planning error, despite the lack of visual reafferences of the moving hand (open loop of the Jump experiment), as expected from similar previous experiments (Goodale et al., 1986; Pélisson et al., 1986; Prablanc and Martin, 1992; Blouin et al., 1995).

## Materials and Methods

### Participants

Seventeen healthy participants took part in the Prism experiment (nine women and eight men), and eighteen healthy participants took part in the Jump experiment (eleven women and seven men). No participant was involved in both experiments. Mean age was 22.4 (±4) years. All participants were right-handed and gave written, informed consent. Both experiments were conducted in accordance with the Declaration of Helsinki and under French law (March 4, 2002) on human participants’ rights, and were based on non-invasive psychophysical tests.

### Apparatus

**FIGURE 1:**
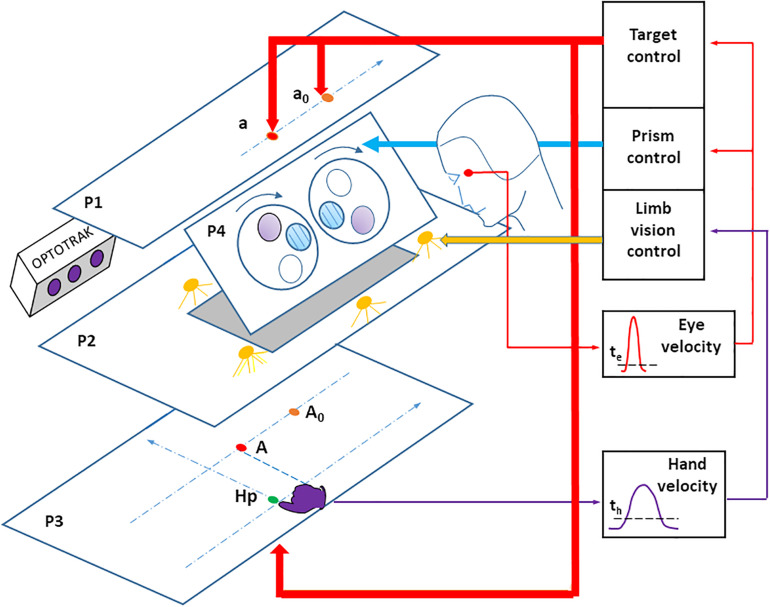
Experimental apparatus. LED targets, placed on the upper stimulation plane (P1) are seen through a half-reflecting mirror (P2) and appear to be placed on the pointing surface (P3). The target A is the mirror image of the target LED a. The pointing hand is only visible when the space between the mirror and the pointing surface is lit using a set of white LEDs. An infrared-emitting diode is attached to the right index fingertip, the position of which is recorded using an OPTOTRAK (3020). A set of Fresnel prisms is placed in front of the eyes, revealing a large visual field (around ± 30 degrees). Prisms are mounted on a motorized disk (lying on plane P4), which allows quick switching from zero to any prism deviation. Eye movements were recorded with a DC EOG amplifier. Online detection of saccade onset (the thin red arrow) was determined by an eye velocity threshold, and used to control the target LEDs (the large red arrow) and fast prism switching (the large blue arrow). Online detection of hand pointing movement onset (the thin violet arrow) was determined by a velocity threshold, and used to control the vision (on/off) of the right hand (the large yellow arrow). For the sake of clarity, the participant’s head has been displaced backward, when in fact the forehead rests against a support on plane P4. Hp: hand starting position. a0 and A0: peripheral target and its image on P3, at the beginning of the prism trial (prism viewing). a and A: peripheral target and its image on P3, after the switch from prism to no-prism viewing. The pointing surface P3 was tilted 17 degrees for comfort. The stimulation plane P1 and the pointing surface P3 were parallel to each other.

The visual stimulus consisted of red, light-emitting diodes (LEDs) located on a plane above the participant’s head in his or her right pointing space (Figure 1). Participants observed targets through a half-reflecting mirror, placed so that the target appeared on a table that the participant pointed to. The pointing surface was black with no reference frame or visual cue. Three LED targets (T1, T2, and T3) appeared along a fronto-parallel line at three locations (30, 130, and 230 mm) to the right of the body axis, and 570 mm away from the participant’s eyes. Finger-to-target masking could not influence the results because the target was a virtual image. A direct view of the pointing hand (through the half-reflecting mirror) could be prevented/allowed by turning off/on a set of white LEDs placed between the mirror and the pointing table. A sagittal red LED (denoted as HP) was used as both a central fixation point, and a starting position for the hand. This fixation target was placed 430 mm from eye level, along the sagittal axis.

A set of motorized Fresnel prisms were placed in front of each eye. Binocular viewing through prisms was controlled by two high-speed stepper motors (Oriental motors PK2913DT, not shown) rotating a Plexiglas disk (290 mm diameter, 2 mm thick). The prisms allowed 20 diopter right or left horizontal deviation (with vision displaced 8 cm to the right or to the left), or null deviation (null prisms are transparent glass with striated lines producing the same, slightly blurred lines as Fresnel prisms). The 57 mm diameter prisms allowed a large field of view (around ± 30°) that included the visual targets, a view of the hand, and visual feedback from the limb.

Horizontal and vertical eye movements were recorded with a calibrated DC EOG amplifier (Prablanc and Martin, 1992). The vertical components, although including eyelid movements, allowed detecting small, horizontal saccade components. Data were sampled at 1000 Hz. Online detection of saccade onset was determined by a 30 deg/s eye velocity threshold, using a two-point central difference algorithm (Bahill and McDonald, 1983) with 10 ms bandwidth. Online detection of saccade onset was used to control target lighting (in both experiments) and fast (<80 ms) online prism switching from 20 right or left diopters to null (during the Prism experiment).

A 3-D hand pointing movements were recorded using an OPTOTRAK (3020) camera at 200 Hz sampling rate. An infrared LED was placed on the tip of the participant’s right index finger. Online detection of pointing movement onset was determined by a fixed 80 mm/s velocity threshold using the same method as for the eye (10 ms bandwidth). This detection was used to control on/off vision of the right hand by turning on/off the white LEDs located by the mirror and the pointing table.

Pointing movements were as natural as possible, with no mechanical constraint or load. In addition, there was full spatial compatibility between stimulus and response, and visual feedback during the pointing movement consisted of the entire limb.

The experiment was fully controlled by a customized program running on a real-time AD-WIN system (Keithley-Metrabyte, Southfield, MI, United States).

### Experimental Design

In both experiments, participants sat comfortably in a chair facing the table, in a dark room. The head was maintained in a fixed position by a forehead rest. Participants were asked to gaze at the fixation target HP and point to it with their right index finger, as long as it was lit. Their right index finger, right hand, and right arm were fully visible. Once the index finger had been within 1 cm^2^ of HP for 1 s, the latter was turned off, and a peripheral target simultaneously appeared at location A. Target A was presented at one of three locations (T1, T2, or T3). It should be noted that only one target was lit at a time. The participant was instructed to look at, and point to target A, as quickly and accurately as possible. They were also asked to avoid making any corrective movements once his/her fingertip had contacted the pointing surface. Finally, they could return to the resting position.

#### The Prism Experiment

A planning error was introduced by displacing the visual scene (hand and target positions) through prisms, prior to movement onset. The Fresnel prisms displaced vision randomly to the right or left. We also introduced “no planning error” trials. Here, null prisms were used and the hand and target were seen without distortion.

**FIGURE 2:**
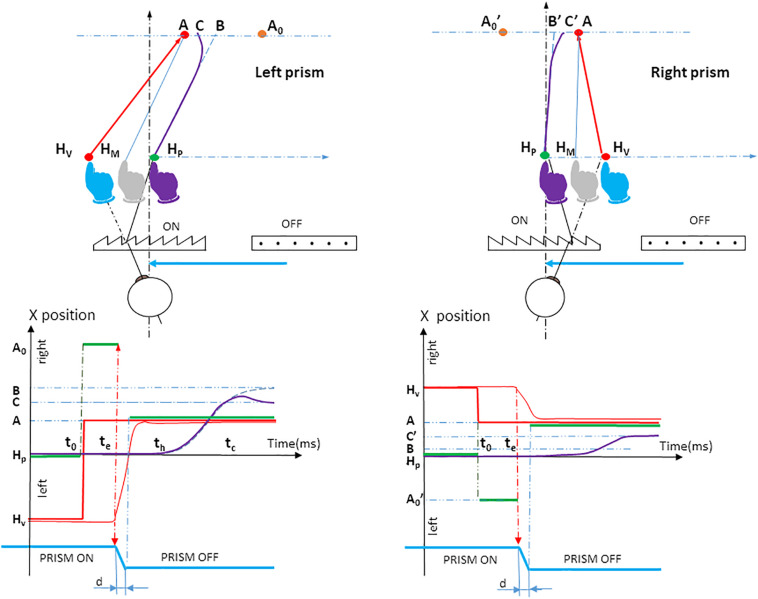
Prism experiment procedure. The upper panel shows the spatiotemporal arrangement of the orthogonal projection of the hand movement onto the pointing plane P3, for left and right prism displacements. The participant initially positioned his or her fingertip at fixation target *H*_*P*_. The physical location of the fingertip is indicated in purple. Both the seen *H*_*P*_ fixation target (red LED) and the seen fingertip (blue) were located on the virtual image *H*_*V*_ due to the visual prism displacement. When the hand (purple) had been maintained on the red fixation target *H*_*P*_ for 1 s, the fixation target was cut off; simultaneously a red peripheral target was turned on at location *A*_0_. Seen through the prism, the target *A*_0_ appears at *A*. The eye saccade was performed along vector *HvA*. At saccade onset, there was a switch from prism to null prism viewing, which occurred simultaneously with a jump of the peripheral target from *A*_0_ to *A* to compensate for the prism removal. During unperturbed trials (null prism), the initial target stimulus was presented at location *A*. The vision of the hand was not distorted. Initially the hand moves toward a point *B* (blue dotted line); this is followed by a correction (bold purple line) that deviates the movement to point *C. BA* represents the actual planning error, taking into account the visual-proprioceptive weighting of the perceived hand position: the gray hand at *H*_*M*_ can be considered as the initial weighted average of prism-distorted vision (blue hand) and unaltered proprioception of the hand (purple hand) seen through the prisms before movement onset. *H_*M*_A* is a theoretical parallel vector to *H_*P*_B*. Dotted blue line: planned hand pointing; Bold purple line: actual hand pointing; *H*_*P*_: the physical fixation target; *H*_*V*_: the seen fixation target; Purple hand: the physical position of the hand before prism onset, and the felt perception of the hand based on proprioception; Blue hand: prism-distorted vision of the hand before movement onset; Gray hand at *H*_*M*_: initial weighted average of vision and proprioception of the hand seen through the prisms before movement onset; *A*_0_, initial peripheral target seen at *A* through the prisms; *A*, peripheral target after prism removal. Note that the participant always perceives the peripheral target in *A*; *B*, the final point of the initially planned movement; *C*, the final point of the movement after correcting for the deviation. The lower panel shows the timing of stimuli and responses. Horizontal green segments indicate different horizontal, physical target locations on the pointing table: *Hp* (seen at *H*_*v*_ until time *t*_0_, which indicates the time at which *Hp* is cut off and *A*_0_ is lit); *A*_0_ from *t*_0_ to *t*_*e*_, which indicates saccade onset (seen in *A*) *A* from *t_*e*__+__*d*_* until trial end (seen naturally), *d* being the duration of prism switching. Thin red, vertical dotted arrows represent the time of prism removal, when simultaneously the target was shifted in order to maintain the view of the peripheral target *A* stationary. As a result, the participant’s view of the target was a simple jump from *H*_*v*_ to *A* (bold red step). At time *t*_*h*_, about 100 ms after saccade onset, the hand was initially planned to move toward a point *B* (blue dotted line), followed by a correction at time *t*_*c*_ (bold purple line) that deviated the movement to point *C*. The (thin blue vertical) arrow *t*_*c*_ is the time of divergence – from the initially planned movement to the corrective movement. Green segments, lateral positions of the physical target; Bold red segments, lateral positions of the seen target; Dotted blue line, (lateral direction of) planned hand pointing; Bold purple line, (lateral direction of) hand pointing; Thin red line, lateral direction of eye saccade; *t*_0_, appearance of the peripheral target *A*; *t*_*e*_, saccade onset; *t*_*h*_, hand movement onset; *t*_*c*_, correction latency; *H*_*P*_, the physical fixation target; *H*_*V*_, the seen fixation target; *A*_0_, the initial peripheral target seen at *A* through the prisms; *A*, the peripheral target after prism removal. Note that the participant always perceives the peripheral target in *A*; *B*, the final point of the initially planned movement; *C*, the final point of the movement after correcting for the deviation.

At saccade onset, a switch from prism to null prism viewing was performed within 80 ms or less (i.e., within saccade duration) and was undetected. Simultaneously, the peripheral target jumped from a0 to a, to compensate for the suppression of the visual displacement due to the prism removal. This allowed the peripheral target to always appear in a stationary position at location A (Figure 2). This correction was not performed with null prisms. The peripheral retinal transition from the hand seen through prisms to the hand seen naturally during saccadic suppression (Matin, 1974) was not perceived, simulating pointing toward a stationary target. In order to prevent participants guessing the trial condition from the noise of the motor, stepper motors moved at saccade onset, even in null Prism trials.

**FIGURE 3:**
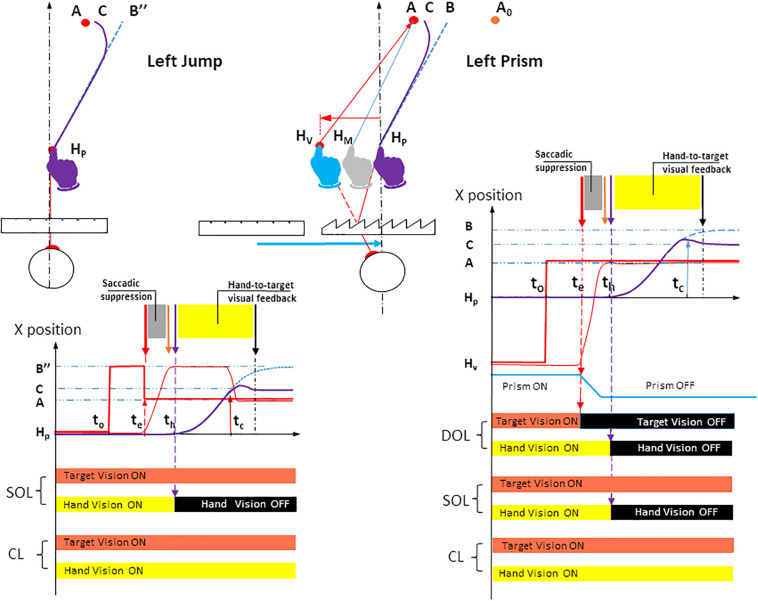
Spatial paths and temporal timings of visuomotor conditions in Jump and Prism experiments for a left deviation. The instructions given to participants were identical in both Jump and Prism experiments: point from the fixation point *H*_*P*_ to the peripheral target as quickly and accurately as possible. Left upper panel: the Jump experiment. After a 1 s fixation on the target *H*_*P*_, a peripheral target appeared in *B*″, while the fixation target was cut off. Saccade onset triggered a target jump from *B*″ to *A*. As a result, the planned hand movement directed toward *B*″ (blue dotted line) caused a planning error *B*″*A*, and was corrected toward *C* (bold purple line). The blue dotted line represents the hand movement toward a stationary target *B*″ (i.e., the planned movement). Jump amplitude *BA* was equal to the mean horizontal planning error *BA* measured in the Prism experiment. The Jump could occur from a point *B*″ located either to the left or right (not represented) of *A*. During stationary trials, the target did not jump, and remained at *B*″. The jump deviation factor consisted of four states: stationary *B*″, *B*″ to *A* jump (for a left jump), and the symmetrical configuration (not represented) for a right jump. Null deviation corresponded to the interpolation of right and left stationary trajectories. Left lower panel: Timing of stimuli and responses for the Jump experiment. Bold red lines represent the seen target stimulus. Bold red segments, lateral positions of the seen target; Dotted blue line, (lateral direction of) planned hand pointing, obtained when *B*″ is stationary; Bold purple line, (lateral direction of) hand pointing with correction; Thin red line, lateral direction of eye saccade; *t*_0_, appearance of peripheral target *B*″; *t*_*e*_, saccade onset, triggering a target jump from *B*″ to *A*; *t*_*h*_, hand movement onset; *t*_*c*_, correction latency; *H*_*P*_, physical fixation target; *B*″, peripheral target before target jump; *A*, peripheral target after target jump; *C*, the final point of the movement after correcting for the deviation. The visuomotor loop factor had two states in the Jump experiment. Single open loop (SOL): target vision was ON throughout the trial and hand vision was ON until hand movement onset *t*_*h*_ (thin dotted purple vertical arrow), then OFF until trial end. Closed loop (CL): target and hand vision were both ON throughout the trial. Right upper panel: Prism. See Figure 1 (upper left) for the meaning of symbols and colors. Prism deviation could be to the left, right (see Figure 1, upper right), or null. Right lower panel: Timings of stimuli and responses for the Prism experiment. The visuomotor loop factor had three states. Double open loop (DOL): target was ON until saccade onset *t*_*e*_ then OFF until trial end, hand vision was ON until hand movement onset *t*_*h*_ then OFF until trial end. Single open loop (SOL): target vision was ON throughout the trial and hand vision was ON until hand movement onset *t*_*h*_ (thin dotted purple vertical arrow), then OFF until trial end. Closed loop (CL): target and hand vision were both ON throughout the trial. Bold red segments: lateral positions of the seen target; Dotted blue line: (lateral direction of) planned hand pointing; Bold purple line: (lateral direction of) hand pointing; Thin red line: lateral direction of eye saccade; t_0_, appearance of peripheral target A; t_*e*_, saccade onset; t_*h*_, hand movement onset; t_*c*_, correction latency. *H*_*P*_, physical fixation target; *H*_*V*_, seen fixation target; *A*, seen peripheral target; *B*, the final point of the movement initially planned; *C*, the final point of the movement after correcting for the deviation. Figure 1 shows that this configuration enables a Prism/Jump comparison for the same erroneous planning (*H_*P*_B* and *H_*P*_B*″) toward A.

The Prism experiment examined three factors: the visuomotor loop, target eccentricity and prism deviation. The visuomotor loop factor included three conditions (Figure 3, right). In the double open loop condition (DOL), the target A was lit off at saccade onset, while hand vision was cut off at hand movement onset. This made it possible to assess initial pointing planning, as there was no visual information from the target that could be compared to proprioceptive information from the hand, once the movement was initiated. In addition, any comparison between visual information from the target and from the hand was not possible. In the single open loop condition (SOL), the target was lit throughout the trial, while hand vision was cut off at hand movement onset. SOL only differed from DOL in that target A was lit throughout the trial. In the closed loop condition (CL), both the target and the hand could be seen throughout the trial. This made it possible to assess the efficiency of natural, undistorted hand-to-target visual feedback in correcting erroneous planning. The target eccentricity factor included the three target locations on the fronto-parallel line (T1, T2, and T3). The Prism condition included three states: left, right and null deviations.

All factors and their states (i.e., 3 × 3 × 3 trials) were randomized. Null deviations (50% of trials) were included to prevent prediction. Randomizing prism deviation across target eccentricity prevented learning or adaptation, which is classically observed under prism-displaced vision. Each type of trial was repeated 12 times, except null deviation, which was repeated 24 times. The Prism experiment thus included 432 trials, divided into four sessions separated by a rest period. The present Prism experiment differs from classical prism adaptation studies in that participants are not exposed to a visual-proprioceptive conflict during hand movement.

#### The Jump Experiment

In this experiment, the planning error was introduced by switching the peripheral target at saccade onset (upper left panel, Figure 3). After a 1 s fixation on point HP, a peripheral target appeared at B″. Saccade onset triggered a target jump from B″ to A, which remained lit throughout the trial. As a result of saccadic suppression, the target jump was not consciously detected (Pélisson et al., 1986; Prablanc and Martin, 1992).

The target randomly jumped to the right, to the left, or remained stationary. Jump amplitude was set to the mean planning error measured in the Prism experiment (DOL condition), with the same initial path (Figure 3). The size of the artificially induced planning error had to be the same in the two experiments. The planning error in the Prism experiment was a function of the initial weighted average of (distorted) vision and (unaltered) proprioception of the hand, seen through the prisms before movement onset, while in the Jump experiment it was equal to the jump amplitude. Therefore, the Prism experiment was performed first in order to assess the planning error, which was expected to be between 20 diopters for null dominance and zero for full dominance. Then, in the Jump experiment, the target jump amplitude that determined the planning error was set to the mean error in the Prism experiment DOL condition.

A key difference in the Jump experiment compared to the Prism experiment was the absence of the DOL condition, as this condition was used to determine the amplitude of the deviation in both experiments. The same null prisms were used in both the Jump experiment and the Prism experiment.

The Jump design included three factors: the visuomotor loop, target eccentricity and jump deviation. The visuomotor loop factor included single open loop (SOL) and closed loop (CL) conditions, which were identical to those in the Prism experiment (i.e., visible target throughout the trial). In the SOL condition, vision of the hand was removed at movement onset, whereas it was available in the CL condition (Figure 3, left). Target eccentricity factor was the same as in the Prism experiment once the movement had been initiated (Figure 3, lower row). Jump deviation had four conditions: left jump, right jump and two stationary, left and right conditions (upper left, Figure 3). The null jump was the linear interpolation of right and left stationary trajectories. All factors and their conditions (i.e., 2 × 3 × 4 trials) were randomized. Each type of trial was repeated 12 times, except the null/stationary deviation, which was repeated 24 times. The experiment thus consisted of 288 trials, divided into two sessions separated by a rest period.

The order of Prism and Jump experiments was not counterbalanced, because the Prism experiment had to be performed first in order to assess the planning error.

Despite its technical complexity, the procedure was simple and designed in such a way that in both Prism and Jump experiments: (1) subjects saw the same single stationary target at location A from target onset until movement end (see Figures 2, 3); (2) the same erroneous planning was introduced; and (3) once the movement was initiated, participants saw the target and their own hand without any distortion. The experimental procedure was inspired by an earlier, simplified version (Revol et al., 2010).

### Data Analysis

Data analysis was conducted with Matlab software. Raw data were filtered at 20 Hz for the XY coordinates of the hand, and at 30 Hz for the horizontal and vertical DC EOG signals, using a Savitzky-Golay second order polynomial filter (Savitzky and Golay, 1964). Hand movement onset and offset used a velocity threshold of 50 mm/s and the eye saccade threshold was 30 deg/s. These thresholds were used by an algorithm to compute the following eye and hand movement parameters. Pointing error was defined as the distance along the X axis between the final pointing position and target distance, as Prism or Jump deviations were only applied along the X axis. It should be noted that a positive error indicates an under-correction of the deviation. Pointing variability was assessed by within-subject standard deviation of final pointing position, which referred to as pointing variable error. Hand movement latency was defined as the difference between hand movement onset and peripheral target onset. Similarly, eye movement latency was defined as the difference between eye movement onset and peripheral target onset. Hand movement duration was computed as the difference between hand movement offset and onset. Correction latency refers to the earliest point at which spatiotemporal parameters began to deviate from reference trajectories (i.e., DOL pointing for Prism perturbation, and pointing to stationary targets for Jump perturbation). The ratio of hand acceleration duration to total hand movement duration was defined as the time elapsed between hand movement onset and peak hand velocity divided by hand movement duration (TPV/movement duration).

The angle of the velocity vector and the horizontal velocity component were computed for each individual trial. Spatial and temporal eye and hand velocity, and velocity vector angle curves were synchronized to average individual trials.

In order to compare Jump and Prism experiments based on the same kinematic features for each deviation factor (left, null, and right), we performed a linear interpolation of the response to a null jump at the location of target A between responses to each of the two stationary targets B″ (Figure 3, upper left) and its symmetrical target (not shown).

**FIGURE 4:**
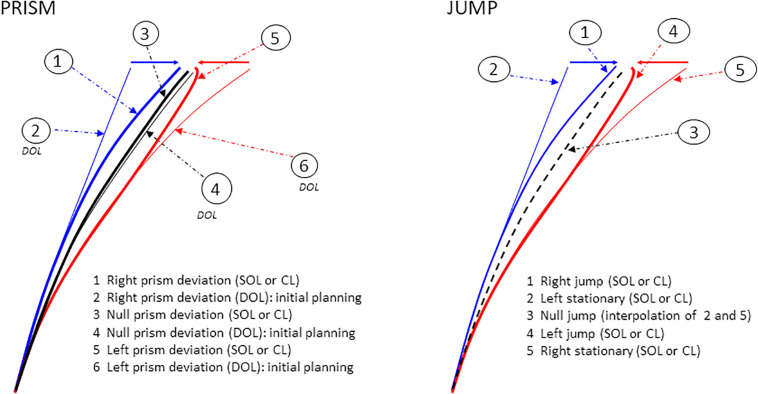
Examples of single hand X–Y trajectories in Prism and Jump experiments. Prism experiment: right (blue), left (red), and null (black) prism deviation. Six hand trajectories are possible. Correction latency refers to the earliest point at which spatiotemporal parameters begin to deviate from reference trajectories. It is measured as the divergence between paths 1 (right prism deviation, SOL or CL) and 2 (initial planning, DOL) for right deviations, and between paths 5 (left prism deviation, SOL or CL) and 6 (initial planning, DOL) for left deviations. Paths 3 and 4 correspond to null prism deviation, under SOL/CL, and DOL respectively. Jump experiment: right (blue), left (red), and null (black) jump deviation. Five hand trajectories are possible. Correction latency corresponds to divergence from reference trajectories, between paths 1 (right jump, SOL or CL) and 2 (left stationary target) for right deviations, and between paths 4 (left jump, SOL or CL) and 5 (right stationary target) for left deviations. Path 3, which corresponds to the null jump, is the linear interpolation of paths 2 and 5. Reference trajectories refer to DOL pointing for Prism perturbation, and to pointing to stationary targets for Jump perturbation.

In addition, statistical analyses were performed to detect the earliest point at which Prism or Jump trajectories began to diverge from reference trajectories (DOL for Prism perturbation, and stationary targets for Jump perturbation, see Figure 4). Individual trials with hand movement peak velocities exceeding 2700 mm/s were excluded from the analysis. For each Prism and Jump experiment, each target eccentricity, each subject, and each (right or left) deviation, the family of repeated trials of the velocity vector (angular and horizontal velocity) for deviated trials was compared to that of repeated trials for reference trajectories. This corresponded to divergence of trajectory 1 from 2, to 5 from 6 for the Prism perturbation, and of trajectory 1 from 2 to 4 from 5 for the Jump perturbation (see Figure 4). A circular parametric Watson–Williams test (Matlab Circular Statistics Toolbox) was run on angular velocity based on a 1 ms step, starting from 120 ms after movement onset until the time when decreasing velocity reached twice the velocity threshold. In order to avoid transient erroneous detections of divergence between the families of velocity vector angles, the circular statistical test had to be significant for at least 70 ms before divergence was validated. When significance fell off, the divergence counter was reset to zero. In addition to angular velocity divergence, a *t*-test was used to measure the divergence of horizontal velocities between deviated and reference trajectory trials. Correction latency was measured as the minimum of the two angular and horizontal velocity divergence measures. When decreasing velocity fell under the double of the velocity threshold, without significant divergence, that time was taken as a default underestimate of correction latency. For both types of perturbation, correction latency was estimated as the difference between the time of divergence, and the time of movement onset (*t*_*h*_).

A four-way repeated measures ANOVA (ANOVA-4) was performed on the main movement parameters in both experiments (pointing constant error, pointing variable error, duration, peak velocity, ratio between acceleration phase and total hand movement duration, correction latency), with perturbation (Prism, Jump) as a categorical, between-subjects factor, and visuomotor loop (open, closed), target eccentricity (T1, T2, and T3) and deviation (Left, Right) as within-subjects factors. It should be noted that open loop refers to the single open loop (SOL) for both Prism and Jump experiments in the “Results” section. The double open loop (DOL) condition in the Prism experiment was not included, as it was only used to determine the actual planning error.

For each Prism and Jump perturbation, a three-way repeated measures ANOVA (ANOVA-3) was performed on each movement parameter, with visuomotor loop (open, closed), target eccentricity (T1, T2, and T3) and deviation (Left, Null, Right) as within-subjects factors.

As a control, and in order to check that subjects behaved in the same way during Prism and Jump experiments, the spatiotemporal characteristics peak velocity (PV), time-to-peak velocity (TPV), duration and pointing error in unperturbed trials (i.e., null Prism, and null Jump deviation) were compared between the two experiments using a three-way ANOVA (ANOVA-3null) with perturbation (Prism, Jump), visuomotor loop (open, closed loop), and target eccentricity (T1, T2, and T3) as factors.

In order to test whether learning occurred, despite the undetected prism displacement or target jump, an ANOVA was performed on pointing errors. This compared the three first, and three last CL perturbed trials for each target, and each right/left deviation.

Finally, statistical tests were performed on eye and hand latencies to check that the same eye-hand planning sequences were observed in both Prism and Jump experiments.

All significance levels were set at 0.05. Where applicable, we used the Greenhouse–Geisser correction to correct for violation of sphericity. The Scheffé *post hoc* test was used when appropriate. Statistical analyses were performed using Statistica^TM^.

## Results

One participant in the Prism experiment was excluded from the analysis because his/her z-score for hand movement PV exceeded two and a half. Overall, eight values were missing for correction latency among a total of 432 trials.

### Participants’ Reports

At the end of all sessions, participants were asked about their experience. All reported seeing a single, stationary target in both experiments. In the Jump experiment, they did not notice the intra-saccadic target jump, but did report a slight inaccuracy when looking at their moving hand. Participants in the Prism experiment did not notice the visual shift of their hand prior to movement onset. They did, however, report feeling abnormally inaccurate when they could see their hand during the movement, and had to intentionally correct it.

### Eye and Hand Latencies

ANOVAs and correlations were run to verify that the same eye-hand planning sequences were observed in both the Prism and the Jump experiments. In both cases, saccade latencies (mean Prism = 242 ms; mean Jump = 220 ms) and hand latencies (mean Prism = 289 ms; mean Jump = 299 ms) were correlated (R Prism = 0.76; R Jump = 0.71; *p* < 0.0001). The small difference in eye-hand latency (47 ms in the Prism experiment and 79 ms in the Jump experiment) indicates that hand motor planning was not influenced by visual updating of the target position at saccade end, as mean saccade duration was about 126 ms for the Prism experiment and 122 ms for the Jump experiment; thus, mean hand movement onset occurred during saccade execution.

In the Prism experiment, an ANOVA-3 performed on hand latency showed significant effects of eccentricity (*F*(2,32) = 10.5, *p* < 0.001) and prism deviation (*F*(2,32) = 9.8, *p* < 0.001), although it should be noted that differences did not exceed 23 ms. The open/closed loop factor was not significant (*p* = 0.97). Saccade latency followed the same trend: no significant effect of open/closed loop (*p* = 0.72); an eccentricity effect (*F*(2,32) = 24.8, *p* < 0.0005); and a prism deviation effect (*F*(2,32) = 4.3, *p* < 0.05). No difference exceeded 20 ms.

In the Jump experiment, an ANOVA-3 performed on hand latency showed a significant effect of eccentricity (*F*(2,34) = 7.1, *p* < 0.01). The open/closed loop factor (*p* = 0.31) and prism deviation (*p* = 0.06) were not significant. No difference exceeded 15 ms. Saccade latency was influenced by the open/closed loop factor (*F*(1,17) = 5.6, *p* < 0.05) although the difference was minimal. There was an eccentricity effect (*F*(2,34) = 5.2, *p* < 0.05), but no significant prism deviation effect (*p* = 0.66). No difference exceeded 15 ms.

### Control Condition (Null Deviation)

For a given path and under natural conditions (null deviation), peak velocity (PV) and time to peak velocity (TPV) are the earliest markers of initial planning, and were analyzed in addition to movement duration, target eccentricity, and pointing error. An ANOVA-3null was run on these variables. For PV, no significant difference was found for the Prism/Jump factor (*p* = 0.21). The open/closed loop factor indicated that feedback speeded-up the movement (*F*(1,33) = 8.1, *p* < 0.01). Target eccentricity (*F*(2,66) = 761, *p* < 0.0001) indicated that PV was a function of movement amplitude. For TPV, neither the Prism/Jump factor (*p* = 0.9), nor the open/closed loop factor (*p* = 0.33), nor target eccentricity were significant (*p* = 0.09).

X (horizontal) pointing errors in the null deviation condition were not significantly different between the Prism and the Jump experiment (*p* = 0.88), and between open and closed loop conditions (*p* = 0.22). There was, however, a large target eccentricity effect (*F*(2,66) = 22.4, *p* < 0.0001). For in-depth (Y) pointing errors, neither perturbation type, open/closed loop, or target eccentricity was significant (all *p* ≥ 0.2). Movement duration was not significantly different between Prism and Jump experiments, or between open and closed loop conditions (all *p* > 0.5), see Table 2. Thus, with the exception of target eccentricity (*F*(2,66) = 38, *p* < 0.001), no factor significantly influenced the acceleration phase of the movement. Overall, this indicates that subjects did not use a task-dependent strategy to optimize their performance.

**TABLE 2:** Means and standard deviations of the principal movement parameters.

**Visuomotor loop**			**Open loop**										**Closed loop**							
**Target**			**T1**			**T2**			**T3**			**T1**			**T2**			**T3**			
**Deviation**			**Left**	**Null**	**Right**	**Left**	**Null**	**Right**	**Left**	**Null**	**Right**	**Left**	**Null**	**Right**	**Left**	**Null**	**Right**	**Left**	**Null**	**Right**
Pointing error	Prism	mean	46	9	33	36	-6	45	31	-8	52	16	0	15	11	0	19	13	-1	13
(mm)		*SD*	17	10	12	13	13	14	15	15	14	23	7	12	17	7	15	14	12	17
	Jump	mean	16	4	9	10	-2	13	5	-7	19	6	0	7	7	0	8	6	0	7
		*SD*	11	7	11	11	10	13	14	12	13	9	3	7	7	4	7	6	5	7
Duration (ms)	Prism	mean	413	411	428	420	402	405	421	402	402	443	411	410	444	389	404	415	397	408
		*SD*	55	52	57	56	53	48	54	55	53	54	49	60	50	49	48	49	46	55
	Jump	mean	414	421	409	407	399	392	399	400	394	440	420	406	418	398	403	408	408	414
		*SD*	63	66	65	51	64	59	60	57	56	47	58	61	51	56	61	58	52	52
TPV/duration	Prism	mean	0,35	0,37	0,37	0,36	0,38	0,38	0,36	0,38	0,38	0,33	0,37	0,41	0,33	0,38	0,39	0,38	0,39	0,39
		*SD*	0,05	0,05	0,04	0,06	0,05	0,05	0,05	0,05	0,04	0,05	0,04	0,05	0,05	0,04	0,05	0,05	0,04	0,04
	Jump	mean	0,41	0,36	0,35	0,39	0,38	0,37	0,40	0,39	0,39	0,41	0,37	0,34	0,39	0,38	0,37	0,40	0,38	0,40
		*SD*	0,05	0,05	0,04	0,06	0,05	0,05	0,05	0,05	0,04	0,05	0,04	0,05	0,05	0,04	0,05	0,05	0,04	0,04
Peak velocity	Prism	mean	1802	1693	1595	1969	1851	1777	2172	2060	1946	1824	1701	1636	2028	1898	1762	2207	2036	1970
(mm/s)		*SD*	310	286	260	296	307	314	321	322	310	317	283	283	328	318	297	332	292	270
	Jump	mean	1621	1499	1483	1849	1744	1707	2064	1961	1929	1632	1519	1510	1871	1775	1726	2085	1979	1912
		*SD*	310	286	260	296	307	314	321	322	310	317	283	283	328	318	297	332	292	270
Correction	Prism	mean	323		370	343		359	362		361	241		257	265		253	321		267
latency (ms)		*SD*	83		58	69		55	43		50	61		65	58		70	44		55
	Jump	mean	154		192	194		156	250		229	176		169	181		161	251		215
		*SD*	53		70	59		35	63		59	39		43	56		33	64		56

### Main Movement Parameters

Table 2 shows means and standard deviation for the principal movement parameters. Table 3 summarizes the results of the four-way analysis of variance (ANOVA-4) with simple interactions.

**TABLE 3:** Summary of the four-way analysis of variance (ANOVA-4), showing simple interactions.

		**Sources of variation**									
		**P**	**L**	**T**	**D**	**P X L**	**P X T**	**P X D**	**L X T**	**L X D**	**T X D**
Pointing error	*F*	51.7	147.4	0.11	1.43	63.8	0.05	0.09	1.97	1.06	24.4
	*p*	<0.0001	<0.0001	0.9	0.2	<0.0001	0.95	0.8	0.16	0.31	<0.0001
Movement duration	*F*	0.25	5	17.2	40.9	0.6	1.2	1.42	0.6	18.8	6.07
	*p*	0.62	<0.05	<0.0001	<0.0001	0.44	0.3	0.24	0.55	<0.001	<0.01
Peak velocity	*F*	1.17	15.6	820	283	1.62	8.16	12.8	0.14	1.39	1.3
	*p*	0.29	<0.001	<0.0001	<0.0001	0.21	<0.001	<0.005	0.87	0.25	0.28
TPV/duration	*F*	1,19	<0.01	15.6	0.5	1.1	0.8	87.9	4.7	18.1	2.9
	*p*	0.28	0.99	<0.0001	0.5	0.3	0.45	<0.0001	<0.05	<0.005	0.06
Correction latency	*F*	121	23.2	22.6	0.58	19	2.7	1.7	0.6	8.49	8.42
	*p*	<0.0001	<0.0001	<0.0001	0.5	<0.0005	0.07	0.2	0.5	<0.01	<0.001

*P, perturbation (Prism/Jump); L, visuomotor loop (Open/Closed Loop); T, target eccentricity (T1/T2/T3); D, deviation direction (Left/Right).*

#### Pointing Error

**FIGURE 5:**
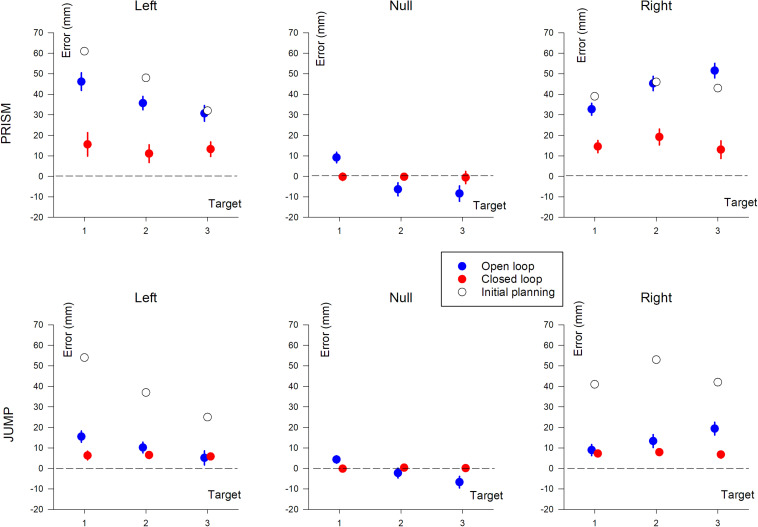
Horizontal pointing error (in mm) based on target position (T1, T2, and T3). The three columns show deviation direction (Left, Null, and Right). Upper row, Prism experiment; Lower row, Jump experiment. Blue, single open loop (SOL); Red, closed loop (CL); Black, initial planning.

Pointing errors are illustrated in Figure 5.

#### Prism vs. Jump

To assess the spatial characteristics of pointing errors, a repeated measures ANOVA (ANOVA-4) was performed on horizontal (X) pointing errors, with perturbation type (Prism/Jump) as a between-subjects factor, and visual feedback (open/closed loop), target eccentricity (T1/T2/T3) and deviation (left/right) as within-subjects factors.

This found an effect of Prism/Jump perturbation (*F*(1,33) = 51.8, *p* < 0.0001), with larger errors in the Prism compared to the Jump condition (see Table 2). The expected effect of visual feedback was observed (*F*(1,33) = 147.4, *p* < 0.0001), with larger errors in the open than the closed loop condition. Overall, errors ranged from 40 mm to 12 mm for the open loop condition for Prism and Jump experiments, respectively, and from 14 mm to 7 mm for the closed loop condition in Prism and Jump experiments, respectively. Target eccentricity was not significant (*p* = 0.89), nor deviation direction (*p* = 0.24). No significant interaction was observed between Prism/Jump perturbation and target eccentricity (*p* = 0.95), nor between Prism/Jump perturbation and left/right deviation (*p* = 0.76).

An interaction was observed between Prism/Jump perturbation and the open/closed loop condition (*F*(1,33) = 63.9, *p* < 0.0001). It was due to a large difference in the correction between Prism/Jump perturbation during open loop pointing. Small, corrected errors were expected to occur during open loop pointing in the Jump experiment, as this is a known phenomenon (Bridgeman et al., 1979; Goodale et al., 1986; Pélisson et al., 1986; Prablanc and Martin, 1992; Sarlegna et al., 2003, 2004).

In closed loop conditions, pointing errors were larger for Prism than for Jump perturbation (*F*(1,33) = 5, *p* < 0.05). Overall, the relative correction was 67% for Prism and 83% for Jump. Neither target eccentricity (*p* = 0.42), nor deviation direction (*p* = 0.41) were significant.

#### Prism Experiment

An ANOVA-3 showed the influence of the open/closed loop on pointing error (*F*(1,16) = 94.8, *p* < 0.0001). No target eccentricity effect was observed (*p* = 0.12). A prism deviation (left, right, or null) effect was found (*F*(2,32) = 31.1, *p* < 0.0001). A *post hoc* Scheffé analysis of prism deviation showed significantly different pointing for left and null prism (no deviation) conditions, and for right and null prism conditions (*p* < 0.0001 for both). Pointing was not significantly different between left and right deviation (*p* = 0.65).

#### Jump Experiment

Pointing error was found to be influenced by the open/closed loop condition (*F*(1,17) = 9.61, *p* < 0.01) and deviation direction (*F*(2,34) = 12.1, *p* < 0.005). No significant effect of target eccentricity was found (*p* = 0.1). A *post hoc* Scheffé analysis of deviation found a significant difference between left and null, and right and null (*p* < 0.005), but no difference between left and right deviation (*p* = 0.6).

#### Pointing Variable Error

##### Prism vs. jump

Visual feedback was found to influence pointing variable errors (*F*(1,32) = 13.2, *p* < 0.001), with larger errors in the open (15 mm) than the closed loop condition (12.4 mm). The Prism/Jump perturbation (*p* = 0.46), target eccentricity (*p* = 0.34), and left/right deviation (*p* = 0.51) were not significant. In closed loop conditions, none of these factors were significant.

##### Prism experiment

The ANOVA-3 showed a target eccentricity effect (*F*(2,30) = 4.3, *p* < 0.05) and a prism deviation (left, right, or null) effect (*F*(2,30) = 5.7, *p* < 0.01). A *post hoc* analysis showed significant differences between targets 1 and 3 (*p* < 0.05), and between left and null deviation (*p* < 0.05). The open/closed loop factor was not significant (*p* = 0.15).

##### Jump experiment

Variable error was a function of the open/closed loop condition (*F*(1,17) = 74.5, *p* < 0.0001), and deviation (*F*(2,34) = 57.2, *p* < 0.0001). Target eccentricity was not significant (*p* = 0.68). A *post hoc* Scheffé analysis of deviation showed a significant difference between left/null and right/null (*p* < 0.0001), but no difference between left and right deviation (*p* = 0.95).

### Hand Movement Duration

**FIGURE 6:**
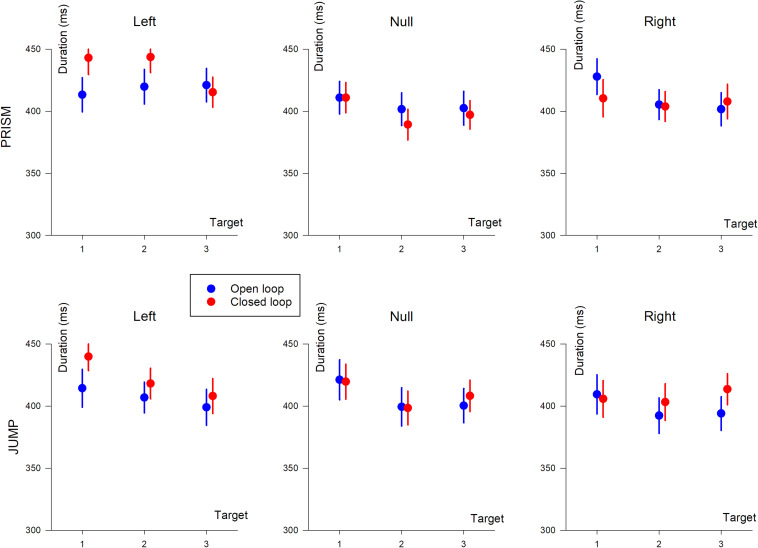
Hand movement duration (in ms) based on target position (T1, T2, and T3). The three columns show deviation direction (Left, Null, and Right). Upper row, Prism experiment; Lower row, Jump experiment. Blue, single open loop (SOL); Red, closed loop (CL).

Hand movement durations are illustrated in Figure 6.

#### Prism vs. Jump

An ANOVA-4 revealed no significant effect of Prism/Jump perturbation on movement duration (*F*(1,33) = 0.25, *p* = 0.6). An effect of open/closed loop was observed (*F*(1,33) = 5, *p* < 0.05), with slightly longer durations in the closed loop (418 ms) than the open loop (409 ms) condition.

Target eccentricity was significant (*F*(2,66) = 17.2, *p* < 0.0001). A *post hoc* analysis showed that movement durations differed between target T1 and the other targets (*p* < 0.0001), with longer durations for target T1. The left/right deviation factor was also significant (*F*(1,33) = 41, *p* < 0.0001), with longer durations for left, compared to right deviations. Furthermore, an interaction was observed between open/closed loop pointing and left/right deviation (*F*(1,33) = 18.9, *p* < 0.001), and between target eccentricity and left/right deviation (*F*(2,66) = 6.1, *p* < 0.01). Target eccentricity and left/right effects were likely to be due to changes in synergies in the elbow-shoulder muscle coupling when pointing to the left or to the right (see change in synergy and reversal of path direction in Figure 2, left column).

#### Prism Experiment

An ANOVA-3 showed that the open/closed loop factor did not influence movement duration (*p* = 0.8). A target eccentricity effect was observed (*F*(2,32) = 8.9, *p* < 0.005). Duration slightly decreased as a function of eccentricity (419, 411, and 408 ms for T1, T2, and T3, respectively). A prism deviation (left, null, or right) effect was found (*F*(2,32) = 24.7, *p* < 0.0001). A *post hoc* analysis showed that hand movement duration differed between left and null prism deviation, and between left and right deviation (*p* < 0.001 for both). On the other hand, duration was not significantly different for null and right prism deviation (*p* = 0.12).

#### Jump Experiment

Movement duration depended on open/closed loop condition (*F*(1,17) = 6.2, *p* < 0.05), with slightly longer duration during closed loop (413 ms) compared to open loop pointing (404 ms). Hand movement duration depended on the deviation (*F*(2,34) = 12.5, *p* < 0.0001) and on the target eccentricity (*F*(2,34) = 26.4, *p* < 0.0001). A *post hoc* analysis showed a significant difference between left/null (*p* < 0.05) and left/right deviations (*p* < 0.001), and no difference between right and null deviation (*p* = 0.12).

### Hand Movement Peak Velocity

#### Prism vs. Jump

An ANOVA-4 revealed that hand movement PV was not a function of the Prism/Jump factor (*p* = 0.29). The open/closed loop factor was, however, significant (*F*(1,33) = 15.6, *p* < 0.001). PV ranged from 1847 mm/s during closed loop pointing, to 1826 mm/s in open loop pointing. PV was a function of target eccentricity, with larger values for more eccentric targets (*F*(2.66) = 821, *p* < 0.0001). Right/left deviation was also significant (*F*(1,33) = 282.9, *p* < 0.0001). Left deviation led to a larger PV than right deviation.

#### Prism Experiment

An ANOVA-3 showed that PV was a function of the open/closed loop factor (*F*(1,16) = 7.9, *p* < 0.05), target eccentricity (*F*(2,32) = 660, *p* < 0.0001), and right/null/left deviation (*F*(2,32) = 140, *p* < 0.0001). No interactions were found.

#### Jump Experiment

PV values were sensitive to the open/closed loop factor (*F*(1,17) = 16.5, *p* < 0.001), target eccentricity (*F*(2,34) = 489, *p* < 0.0001) and deviation (*F*(2,34) = 89, *p* < 0.0001). Once again, no interactions were found.

### Ratio Between Acceleration Phase and Total Hand Movement Duration: TPV/Duration

#### Prism vs. Jump

The relative symmetry of hand movement acceleration and deceleration phases was analyzed as the ratio of the acceleration phase and hand movement duration (i.e., TPV/duration). An ANOVA-4 showed a significant effect of target eccentricity (*F*(2,66) = 15.6, p < 0.0001). A *post hoc* analysis revealed that target T3 dominated the eccentricity factor. Prism/Jump (*p* = 0.28), open/closed loop (*p* = 0.99) and Right/left deviation (*p* = 0.5) factors were not significant.

#### Prism Experiment

An ANOVA-3 showed that TPV/duration was a function of target eccentricity (*F*(2,32) = 5.6, *p* < 0.01) and of right/null/left deviation (*F*(2,32) = 30.7, *p* < 0.0001). *Post hoc* analysis found significant differences between targets 1 and 3 only, and between the three deviations except null and right. Open/closed loop factor was not significant (*p* = 0.55), with a ratio around 0.37.

#### Jump Experiment

The TPV/duration ratio was sensitive to target eccentricity (*F*(2,34) = 9.4, *p* < 0.005) and to deviation (*F*(2,34) = 43.2, *p* < 0.0001) factors. *Post hoc* analysis showed that target T3 dominated the eccentricity factor and significant differences between the three deviations except null and right. Open/closed loop factor was not significant (*p* = 0.81).

### Correction Latency

**FIGURE 7:**
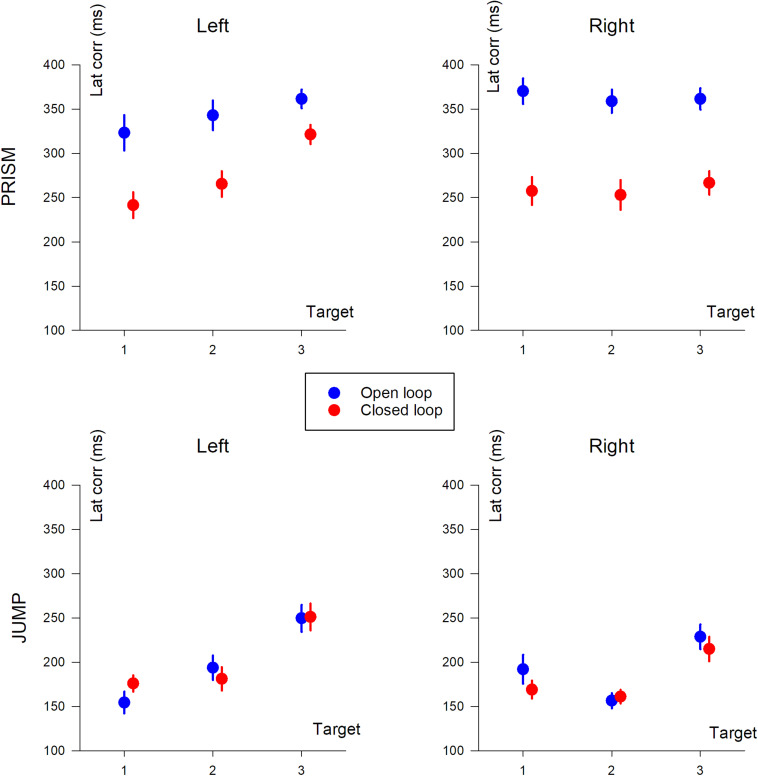
Correction latency (in ms) based on target position (T1, T2, and T3). The two columns show deviation direction (Left and Right). Upper row, Prism experiment; Lower row, Jump experiment. Blue, single open loop (SOL); Red, closed loop (CL). Mean data, including participants with missing data.

Correction latency is the time it takes to modify the trajectory in response to the perturbation. In our experiment, it was defined as the time interval between movement onset, and statistical signs of divergence between reference trajectories and perturbed trajectories, either with (CL) or without (SOL) feedback. Correction latencies are illustrated in Figure 7.

#### Prism vs. Jump

The same ANOVA-4 applied to duration, pointing error, PV, and TPV/duration was applied to correction latency. It showed a significant effect of Prism/Jump perturbation (*F*(1,28) = 121.8, *p* < 0.0001). Correction occurred later for Prism (310 ms) than for Jump perturbation (194 ms). The open/closed loop factor was significant (*F*(1,28) = 23.3, *p* < 0.001). Correction latency was shorter in the closed loop (230 ms) than the open loop (274 ms) condition. Target eccentricity was also significant (*F*(2,56) = 22.6, *p* < 0.0001), while left/right deviation was not (*p* = 0.45). A *post hoc* analysis showed that target T3 dominated the eccentricity factor with respect to correction latency.

There was a strong interaction between Prism/Jump and open/closed loop factors (*F*(1,28) = 19.06, *p* < 0.001). Correction latency ranged from 353 ms to 196 ms in Prism and Jump open loop conditions, respectively, and from 268 ms to 192 ms in Prism and Jump closed loop conditions, respectively. For jump, there was no difference in latency between open and closed loop, while for prism the latency was longer for the open loop than for the closed loop.

#### Prism Experiment

An ANOVA-3 revealed an open/closed loop effect (*F*(1,14) = 24.7 *p* < 0.001), a target eccentricity effect (*F*(2,28) = 4.89, *p* < 0.05), but no significant deviation effect (*p* = 0.7).

#### Jump Experiment

Neither the open/closed loop effect was significant (*p* = 0.6), nor left/right deviation (*p* = 0.2). However, target eccentricity was significant (*F*(2,34) = 21.6 *p* < 0.0001).

### Hand Trajectory

**FIGURE 8:**
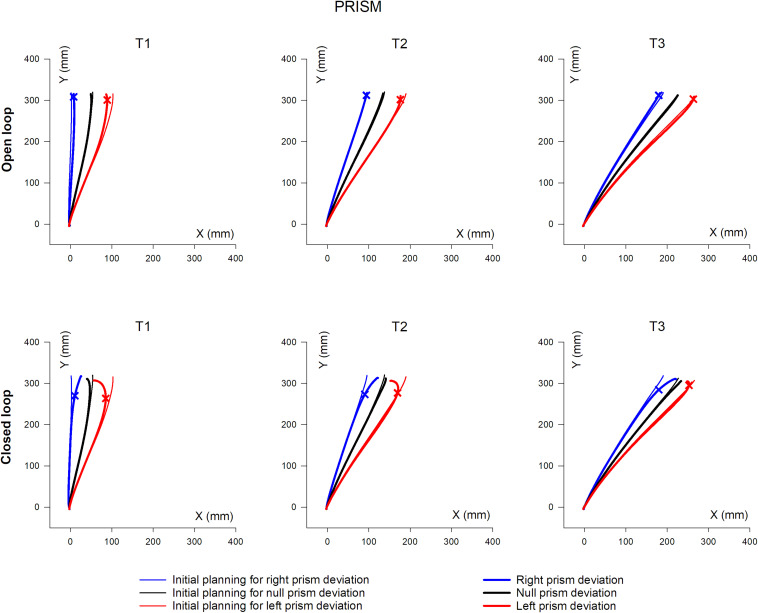
Overall, mean XY hand paths of the pointing movement in Prism experiments, in single open loop (SOL) or closed loop (CL) conditions. The three columns represent target eccentricities T1, T2, and T3. In the Prism experiment, thin lines represent the double open loop pointing (DOL) and reflect the erroneous initial planning, in the case where a leftward (red) or rightward (blue) prism deviated the hand position prior to movement onset. Thick lines represent corrected trajectories either with (CL) or without (SOL) vision of the hand. In the Jump experiment, thin lines represent reference trajectories to stationary targets (i.e., initial planning), while bold lines correspond to corrected trajectories, in the condition where the target jumps at saccade onset either leftward (red) or rightward (blue), prior to hand movement onset (see also Figure 1 and Table 2). Bold lines correspond to either the single open loop (SOL) or closed loop (CL) for both Prism and Jump experiments. Crosses indicate the earliest point of significant hand path divergence (in either the SOL, or in the CL condition).

**FIGURE 9:**
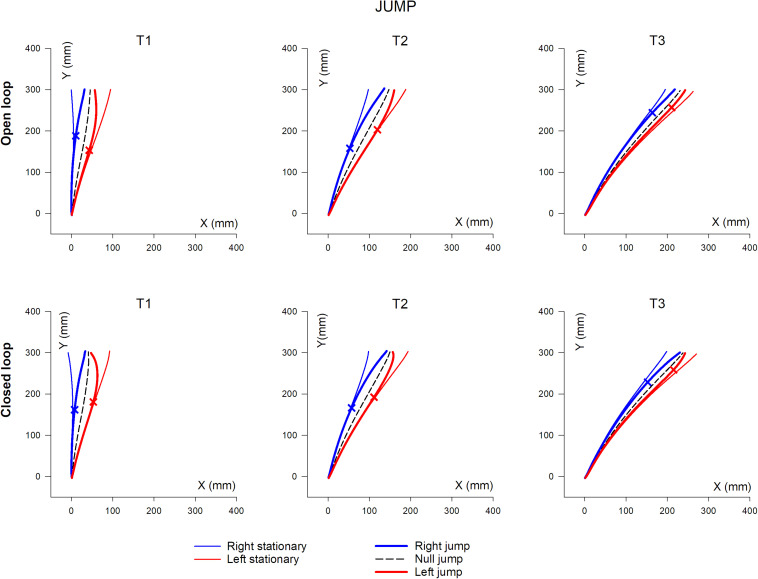
Overall, mean XY hand paths of the pointing movement in Jump experiments, in single open loop (SOL) or closed loop (CL) conditions. The three columns represent target eccentricities T1, T2, and T3. In the Prism experiment, thin lines represent the double open loop pointing (DOL) and reflect the erroneous initial planning, in the case where a leftward (red) or rightward (blue) prism deviated the hand position prior to movement onset. Thick lines represent corrected trajectories either with (CL) or without (SOL) vision of the hand. In the Jump experiment, thin lines represent reference trajectories to stationary targets (i.e., initial planning), while bold lines correspond to corrected trajectories, in the condition where the target jumps at saccade onset either leftward (red) or rightward (blue), prior to hand movement onset (see also Figure 1 and Table 2). Bold lines correspond to either the single open loop (SOL) or closed loop (CL) for both Prism and Jump experiments. Crosses indicate the earliest point of significant hand path divergence (in either the SOL, or in the CL condition).

Mean hand trajectory in the Prism and Jump experiments are plotted in Figures 8, 9.

### Testing for a Learning Effect

A comparison of horizontal pointing errors for the three early and three late trials for each target showed that none of the factors (Prism/Jump, *p* = 0.12; early/late, *p* = 0.26; target eccentricity, *p* = 0.21; left/right, *p* = 0.34) was significant, indicating that learning did not occur during our experiments.

## Discussion

### Sensory Prediction Is Crucial for Online Correction

Overall, these results are a convincing argument for the critical role of prediction in the automatic control of corrections to inaccurate motor planning. When the predicted reafferences were accurate (the Jump experiment), early and automatic corrections of the planning error were observed, even in the absence of visual feedback from the moving hand. When they were erroneous (the Prism experiment), late and incomplete corrections of the planning error were observed, even when natural visual feedback from the moving hand was available. It should be noted that, once the movement had started, Jump and Prism closed loop conditions were identical – i.e., natural vision of both veridical hand and upper limb, and target. The sole difference was the alteration of prediction about one’s own movement state. It clearly indicates that early, automatic corrections rely upon predictions of visual reafferences of the movement.

### Processing of Visual Feedback in the Absence of Accurate Predictions Is Slow and Voluntary

The role of hand visual feedback (either hand-to-target retinal error, or hand visual-to-proprioceptive error) in movement correction has been extensively investigated (Keele and Posner, 1968; Elliott et al., 2010, 2017; Sarlegna and Mutha, 2015). In order to be complete, feedback processing of planning errors induced by a large, perceived target jump can be time-consuming. Although many studies have identified short correction latencies (100–150 ms) in response to a target jump (Soechting and Lacquaniti, 1983; Brenner and Smeets, 1997; Fautrelle et al., 2010; Smeets et al., 2016; Zhang et al., 2018; Gu et al., 2019), few have paid attention to total movement completion time. When the goal achievement requires a significant change in multi-joint synergies, total movement duration has been found to increase substantially (by over 100 ms, Grea et al., 2000), suggesting that movement completion occurs at later stages (Gaveau et al., 2014). No such increase in total movement duration has been observed in the unconscious target jump paradigm, when the stimulus is displaced at hand movement onset and is undetected by the subject. Consistent with previous studies (Pélisson et al., 1986; Prablanc and Martin, 1992), Oostwoud Wijdenes et al. (2011) found a correction latency of 100 ms for pointing to a small target jump, with no increase in total movement duration. Such a behavior was observed in our study (Jump) with no clear increase between perturbed and unperturbed movement durations.

In our Prism experiment, corrective movements were relatively late and their amplitude reduced despite visual feedbacks of the hand and the target during movement execution. These results suggest that altered prediction is unlikely to be updated by natural visual feedback after movement onset. These late corrections are likely to be the result of conflicting inputs coming from erroneous prediction and natural veridical feedback.

The study by Balslev et al. (2007) suggests that, in addition to accurate prediction, rapid processing of visual feedback errors relies on an intact proprioceptive signal. The authors showed that partial proprioceptive deafferentation, through rTMS, increased latency in initiating a motor correction in response to a visual perturbation in hand position, but not to a target jump.

Other studies have suggested a prism-induced disfacilitation of fast visual feedback processing of natural reaching movements. In a classical pointing task, under moderate prism displacement (10 to 11 degrees), and without being able to see the movement, Redding and Wallace (2004) found movement correction of about 50% in their first trials, which could only be accounted for by prism-induced asymmetry in the structured visual field. Similarly, Veilleux and Proteau (2015) observed, in their first trials of prism exposure, the same 50% error correction in the absence of vision of the movement; however when there was a full view of the movement, they observed a 20% uncorrected error in the first five trials – despite the long movement duration (850 ms). Redding and Wallace (2006) compared first trial errors with two types of visual feedback: continuous, with a full view of the hand during the movement; or terminal with a view of the fingertips at the end of the movement. While final error in the terminal feedback condition was about 48%, it was still about 42% in the continuous feedback condition. This indicates the weak contribution of visual feedback to error correction, prior to adaptation or learning. In these classical prism paradigms, both biased prediction of movement reafferences, and visuo-proprioceptive conflict, could have prevented the fast processing of visual feedback. This, however, was not the case in our Prism experiment, as participants experienced natural visual feedback once the movement had started, without any visuo-proprioceptive conflict.

The finding that corrective latency was 140 ms longer in the Prism compared to the Jump experiment is likely to correspond to a voluntary correction, with a significant residual error. Participants reported that they experienced movement inaccuracy in the Prism experiment, and the need to make a voluntary correction. In the Jump experiment, however, subjects did not report any feeling of inaccuracy, and planning error correction occurred early in both open and closed loop conditions. Pointing was, however, obviously more accurate in the closed than in the open loop condition.

### Sensory Prediction Enables Fast Online Corrections of Movement

In the Jump experiment, a fast, large automatic correction was observed, even when the hand was not visible during the movement (SOL), as classically reported in the literature (Bridgeman et al., 1979; Goodale et al., 1986; Prablanc and Martin, 1992; Bard et al., 1999; Sarlegna et al., 2003). This result is in line with the role of efferent copy in the amendment of rapid corrections before any peripheral sensory feedback processing (Scott, 2004). The error signal may be derived either from a delayed comparison between the predicted sensory feedback (i.e., the output of the forward model) and the actual feedback (Diedrichsen et al., 2005), or from an instantaneous comparison of the goal representation, and the expected sensory feedback (Desmurget and Grafton, 2000). Pointing errors were reduced by visual feedback (CL), without a substantial increase in movement duration (<10 ms). However, correction latencies were similar to those observed without visual feedback (SOL), suggesting that it was not a prerequisite to trigger the corrective process.

We cannot rule out the role of proprioceptive feedback in the online correction of movement between the seen target (throughout the movement) and hand proprioception, as hand and whole limb reafferences were unaltered. However, in the Prism/SOL condition the hand was not visible during the movement, and there was nearly no correction. These observations indicate that if visual-to-kinesthetic error played a role in the automatic, online control of the goal-directed movement, it was insufficient to amend the movement.

### Merging Multisensory Signals for Sensory Prediction

Automatic, online corrections rely on predictions of visual reafferences, based on a reliable internal representation of the hand. The prediction of limb movement visual reafferences relies upon the initial, combined, visual and proprioceptive inputs of the hand before the movement begins. In our experiment, the initial sensory weighting lay roughly mid-way between visual and proprioceptive contributions.

Rossetti et al. (1995); Sober and Sabes (2003) found that unexpectedly shifting the viewed hand prior to movement onset produced a planning bias. The latter corresponded to a visual-proprioceptive weighting, which is in agreement with neurophysiological and modeling approaches that emphasize the role of multisensory fusion for accurate spatial limb representation (Meredith and Stein, 1986; van Beers et al., 1996, 1999; Wallace et al., 1996; Graziano et al., 2000; Ladavas and Farne, 2004). In addition to these behavioral studies, Mulliken et al. (2008) found neuronal encoding representing a forward estimation of the movement state in the posterior parietal cortex (PPC). The merging of these multisensory and efferent signals within the PPC is likely to be responsible for gating the automatic, low-level processing of sensorimotor control. Both a PPC lesion (Pisella et al., 2000) and a TMS perturbation of the PPC of healthy subjects (Desmurget et al., 1999) have been found to disrupt the automatic correction of planning errors. This is in contrast to fronto-striatal lesions which, although they considerably increase the reaction time to initiate a movement, do not disrupt fast automatic corrections (Desmurget et al., 2004). It should be noted that spatial compatibility is a prerequisite for automatic online corrections, as they are selectively suppressed when the required response is incompatible with the stimulus location, such as in anti-pointing (Gritsenko and Kalaska, 2010).

### The Role of Sensory Prediction in Our Awareness of Action

Following the work of Jeannerod, authors have begun to discuss the role of predicted reafferences in motor consciousness (for a review, see Blakemore and Frith, 2003). The forward model enables the CNS to predict the sensory consequences of motor commands, which can be compared to actual reafferences. The CNS can determine whether actual reafferences are compatible with predicted reafferences, and differentiate self-produced from externally produced motor events, contributing to our sense of agency (Wolpert and Ghahramani, 2000; Blakemore and Frith, 2003). Citing the work of Jeannerod (Fourneret and Jeannerod, 1998), and neurophysiological and kinematic studies (Libet, 1983; Haggard and Magno, 1999), Blakemore and Frith (2003) propose that we are aware of the intended, rather than the actual, movement. The role of prediction, based on efference in oculomotor control has been known since the 1950s. Our study contributes to extend this knowledge to limb motor control.

## Data Availability Statement

The raw data supporting the conclusions of this article will be made available by the authors, without undue reservation.

## Ethics Statement

Ethical review and approval was not required for the study on human participants in accordance with the local legislation and institutional requirements. The patients/participants provided their written informed consent to participate in this study.

## Author Contributions

A-EP, PR, OS, CP, and VG designed the experiments. A-EP, CP, and VG ran the experiments and analyzed the data. A-EP and CP created the graphics and wrote the manuscript. All authors reviewed the manuscript, provided suggestions, and approved the final version of the manuscript for publication.

## Conflict of Interest

The authors declare that the research was conducted in the absence of any commercial or financial relationships that could be construed as a potential conflict of interest.
